# A tool for assessing sex/gender bias in epidemiological studies of occupational health: Pilot testing on studies of sedentary behaviour at the workplace and cardiometabolic health

**DOI:** 10.1371/journal.pone.0324391

**Published:** 2025-05-29

**Authors:** Michaela Prigge, Janice Hegewald, Kathrin Reichel, Eva Backé, Karla Romero Starke, Andreas Seidler, Ute Latza

**Affiliations:** 1 Federal Institute for Occupational Safety and Health (BAuA), Unit 3.1 Prevention of Work-related Diseases, Berlin, Germany; 2 Federal Joint Committee (G-BA), Berlin, Germany; 3 Institute and Policlinic for Occupational and Social Medicine (IPAS) of the Medical Faculty of the TU Dresden, Dresden, Germany; Indian Institute of Technology Madras, INDIA

## Abstract

**Background:**

The perspective of sex/gender bias is often missing in tools used to assess study risk of bias in systematic reviews. The aim was to pilot a checklist using an aetiological occupational health research question regarding the impact of sedentary behaviour at the workplace and cardiometabolic health. The checklist examined whether the consideration of sex/gender was associated with different study characteristics.

**Methods:**

A sex/gender checklist developed based on a synopsis of existing instruments with input from the Cochrane Sex/Gender Methods Group was adapted for the present study. This checklist comprises four categories: 1. “Background and conceptual considerations” (3 items), 2. “Study design” (2 items), 3. “Study procedures of investigation or intervention and statistical analysis” (2 items), and 4. “Presentation and interpretation of findings” (3 items). Two independent reviewers evaluated all included studies. Bivariate and multivariable logistic regression analyses were used to examine the consideration of sex/gender across study designs, years of publication, and risk of bias levels (based on the Scottish Intercollegiate Guidelines Network [SIGN]).

**Results:**

Of the 49 studies evaluated with the checklist, none provided detailed information, 69% (n = 34) provided basic information and 31% (n = 15) no information for the consideration of sex/gender. No intervention study provided information for the first two categories. In the third category, all intervention studies (n = 17) and case-control studies (n = 5) provided basic information on sex/gender, while two of the 23 cohort studies did not. In the fourth category, detailed information was found for all study designs (n = 8). Bivariate analyses revealed no association between the consideration of sex/gender and the year of publication (OR per year = 0.89; 95% CI: 0.65, 1.23). A low risk of bias level was not associated with consideration of sex/gender (OR = 0.60; 95% CI: 0.14; 2.50). Compared to intervention studies the odds of considering sex/gender was increased by a factor of 3.6 (95% CI: 1.0, 12.8) in observational studies.

**Conclusion:**

The adapted checklist was applicable to assess the consideration of sex/gender in all studies. None of the primary studies considered sex/gender perspectives in all of the four categories. Further optimisation of the sex/gender tool seems warranted, based on further research on weighting individual categories or items and application of the checklist for occupational epidemiology in general.

## Introduction

The main goal of systematic reviews is the synthesis of evidence on a topic. While synthesising the results, it is imperative to assess of how bias in the primary studies may have affected the overall results [1]. There are a number of tools that evaluate the risk of bias and quality in occupational epidemiological studies with different study designs [2,3]. However, for the most part, bias resulting from insufficient consideration of sex/gender is not taken into account by these instruments.

Sex is defined as the biological differences between men and women, while gender includes the view of roles and relationships that societies attribute to men and women [4,5]. Up to now, it is mainly the biological sex that is surveyed in epidemiological studies and gender-specific aspects are usually not considered. Thus, since 2010, a “two-step approach” is increasingly being applied, where first the biological sex is asked, followed by a survey of the gender identity affiliation [6].

It is important to consider sex and gender in health research because biological and psychosocial differences may result in divergent risks [7,8] and prevention approaches may need to be customised for men and women [4]. Failure to consider sex/gender could lead to significant omissions in these areas that might otherwise go unnoticed. This requires, that not only the binary understanding of sex/gender is included [6]. The risk of unfavourable health consequences can thus be minimised if studies take sex/gender into account [4]. In 2016, the Sex and Gender Equity in Research (SAGER) guidelines were published [9]. The recommendations were based on the work of a panel of 13 experts from nine countries, and state that data should be routinely presented disaggregated by sex and gender. Sex- and gender-based analyses should be reported regardless of positive or negative outcomes [9].

While some studies have described how sex/gender bias can be avoided in epidemiological studies [10,11], only a few bias assessment tools consider the evaluation of these sex/gender-specific aspects. Doull et al., for example, developed an appraisal tool that primarily evaluates sex/gender-specific aspects of systematic reviews and meta-analyses [4,12]. Briefing notes were developed as a communication tool to inform reviewers where sex/gender should be considered in a systematic review (e. g., background or methods) [4]. Suitable instruments for assessing sex/gender bias in primary studies are available only to a limited extent [8,11,13–15]. Often these tools are not suitable for the assessment of primary studies, as they primarily aim at helping with the design of studies [11] or to serve as examples of (good) sex/gender-sensitive practice [8]. Risk of bias tools (Cochrane ROB-2, SIGN, etc) assess internal validity. That is, they consider sources of bias that could impact the reported effect estimates for the entire study sample (e. g., randomization, allocation, recall bias) [16]. For example, SIGN included criteria for selection of subjects, assessment, confounding and statistical analysis to assess the internal validity of observational studies [17]. Thus, these tools consider sex/gender at most as a confounder and do not explicitly address potential differences in how study methods, such as recruitment, exposure measurements, and outcome detection may be impacted by sex/gender [16]. Thus, this sex/gender checklist addresses these gaps.

Demetriou et al. [13] and colleagues collaborated with international experts, including members of the Cochrane Sex/Gender Methods Group, to develop a practicable sex/gender bias checklist for assessing intervention studies in systematic reviews addressing physical activity and sedentary behaviour outcomes in children and adolescents [13–15]. However, sedentary behaviour or lack of physical activity are also problems in working adults. Based on a meta-analysis Prince et al. [18] found that approximately 60% of employees spend their working and waking time in a sedentary posture [18]. In Germany, approximately 50% of employees are mainly sitting during work [19]. Prolonged sitting is associated with different unfavourable health outcomes, including risks for the cardiovascular system and metabolism, resulting in an increase in diseases (such as hypertension and type II diabetes mellitus) and associated with premature mortality [20].

It is important to consider sex/gender effects in studies examining the association between sedentary behaviour and cardiovascular disease. The Cardiovascular Clinical Study Group describes differences in cardiovascular diseases (e. g., ischaemic heart disease) between men and women. For example, diabetes is associated with a greater increase in coronary artery disease risk in women than in men [5]. Men, on the other hand, suffer acute coronary heart syndrome or myocardial infarction more often than women [5]. However, mortality 30 days after an acute coronary syndrome is greater for women (9.6% in women and 5.3% in men) [21]. Men and women also differ with regard to sedentary behaviour, with approximately 50% of men and 34% of women spending more than 75% of their time sitting at work [22].

A systematic review by Reichel et al. [23] of occupational sedentary behaviour and its association with cardiovascular and metabolic diseases, cardiometabolic mortality, or changes in cardiometabolic risk markers narratively described whether the results differed for men and women [23]. However, no suitable instrument was available to assess sex/gender bias in the primary studies.

Thus, our first aim was to adapt the sex/gender checklist for sedentary behaviour as an outcome developed for children and adolescents [13,14] by modifying the checklist for aetiological occupational health questions and observational study designs with sedentary behaviour as an exposure.

The second aim was to determine whether the consideration of sex/gender identified by this tool was associated with the studies’ results using the following research questions:

1. Is the consideration of sex/gender increasing over time in the published research identified?
2. Do studies with a high internal validity (low risk of bias assessed with the SIGN) have a greater consideration of sex/gender?
3. Is the consideration of sex/gender associated with the study design?

## Methods

Demetriou et al. (2019) developed a sex/gender checklist in collaboration with international experts, scientists in this field and members of the Cochrane Sex/Gender Methods Group [13,14]. This checklist was used as a basis and adapted for the research question of the systematic review of Reichel et al. [23].

### Sex/gender checklist

The sex/gender checklist consists of four categories that follow the structure of study publications:

1. “Background and conceptual considerations”,
2. “Study design”,
3. “Study procedures of investigation or intervention and statistical analysis”, and
4. “Presentation and interpretation of findings”.

Each category consists of two or three items, respectively. The items of the checklist are described and explained with questions and further statements (Table 1).

**Table 1:** Adapted sex/gender checklist with specifications supplied by the authors.

Category	No.	Item	Definition	Evaluation category			
				Basic	Detailed	No information provided	Not applicable
Background and conceptual considerations	1	Definition and use of sex and/or gender terminology	Is the use of sex and/or gender terminology defined in the study? Are sexes/genders defined in the text?	Sex/gender is defined in the text (not only sample description, see 6a). Sex and/or gender terminology are not used interchangeably.	Sex and/or gender is defined and used consistently separated or sex and gender have been defined as related concepts that are inextricable and the term sex/gender is used consistently.	No information is provided about the definition and use of sex and/or gender terminology.	Item is not appl.
	2	Sex/gender background information regarding the research question (e. g., prevalence, strength of association)	Is sex/gender background information regarding the research question taken into account? Is it described how the research question affects sex/gender or is it justified if not?	Sex/gender considerations regarding the research question/ **exposure to physical activity/ sedentary behaviour are described.**	Sex/gender considerations regarding the research question/outcomes are described and it is further discussed where sex/gender differences and/or similarities result from.	No information is provided about sex/gender background information on the research question.	Item is not appl.
	3	Theoretical and/or conceptual linkages with sex/gender	Is sex/gender linked up with the theory/concept of the investigation or intervention?	An underlying theory of the intervention is reflected regarding sex/gender.	An underlying theory of the intervention is reflected regarding sex/gender and its use in the intervention planning and/or delivery is described.	No information is provided about a linkage between the intervention theory/concept and sex/gender.	Item is not appl.
Study design	4	Measurement instruments	Are the instruments valid and reliable for sex/gender groups? If data are self-rated: Was a gender specific misclassification considered?	Instruments are used that are not developed for sex/gender groups (reliable or valid) and reasons for this decision are given.	Assessment instruments are used that are developed and/or tested for sex/gender groups (reliable and valid).	No information is provided about the use of valid and/or reliable measurement instruments regarding sex/gender groups.	Item is not appl.
	5	Study sample recruitment	Is the necessity of sampling for sex/gender taken into account, e. g., representativeness of population regarding sex/gender?	It is described how sex/gender was taken into account during sampling (representativeness).	Statistical power calculations for testing hypothesis with respect to sex/gender have been considered.	No information is provided about consideration of sampling regarding to sex/gender.	Item is not appl.
Study procedures of investigation or intervention and statistical analysis	6a	Study population, sample description (classification and %)	Is it described how the study population was classified by sex/gender and distribution of m/f in %?	Sex/gender %m/f of study population sample is described.	Basis for sex/gender classification and %m/f of study population sample is described.	No information is provided about study population, sample description (classification and %)	Item is not appl.
	6b	Study procedures **(assessment of occupational exposure** and/or intervention content and delivery, if appl.)	Are study procedures performed inclusive for sex/gender?	Study procedures are described in terms of sex/gender-inclusiveness.	Study procedures are described in terms of sex/gender-inclusiveness and their sex/gender inclusive implementation is stated.	No information is provided about sex/gender-inclusive study procedures.	Item is not appl.
	7	Statistical modelling and analyses	Statistical analysis takes into account sex/gender differences and possible **gender dependent covariates**^**1**^ analyses are stratified by sex/gender or adjusted for sex/gender and tested for effect modification.	Analyses are stratified by sex/gender or adjusted for sex/gender^2^ and tested for effect modification in statistical analysis.	Statistical analyses with respect to sex/gender have been considered: The statistical analysis takes possible **gender dependent covariates**^**1**^ into account.	No information is provided about consideration of statistical analysis to sex/gender.	Item is not appl.
Presentation and interpretation of findings	8	Participant flow	Is a participant flow chart provided that takes sex/gender into account (e. g., for intervention studies according to the CONSORT Statement (eligibility, estimation of sample size (baseline), dropout rates (post-test, follow-up)?	Sex/gender sample size is provided for baseline.	Sex/gender sample size is provided at all measurements points (e. g., baseline, post-post-test).	No information is provided about the intervention sex/gender sample size at any measurement point (baseline, post-test, follow-up).	Item is not appl.
	9	Statistical results	Is sex/gender presented in tables and figures? Are sex/gender differences and/or similarities described regarding the outcomes?	Descriptive analyses for sex/gender differences and/or similarities are shown regarding the main outcomes.	Descriptive analyses for sex/gender differences and/or similarities are shown regarding the main outcomes and further statistical analyses were carried out to identify possible sex/gender effects (e. g., sex disaggregated analyses, stratified analyses, interactions).	No information is provided about sex/gender similarities and/or differences in the outcomes of the study.	Item is not appl.
	10	Discussion	Are the findings reflected with respect to sex/gender, e. g., representativeness of sex/gender in study population in adequate measure?	The findings are reflected with respect to sex/gender.	The findings are reflected with respect to sex/gender and future directions to sex/gender interventions are discussed.	No reflection is provided about the study findings in respect to sex/gender.	Item is not appl.
**Summary Score**							
Category	1-3	Background, and conceptual considerations	☐ basic/ ☐ detailed/ ☐ no information is provided regarding sex/gender aspects. ☐ Not applicable				
	4-5	Study design	☐ basic/ ☐ detailed/ ☐ no information is provided regarding sex/gender aspects. ☐ Not applicable				
	6-7	Study procedures and statistical analysis	☐ basic/ ☐ detailed/ ☐ no information is provided regarding sex/gender aspects. ☐ Not applicable.				
	8-10	Presentation and interpretation of findings	☐ basic/ ☐ detailed/ ☐ no information is provided regarding sex/gender aspects. ☐ Not applicable				
	1-10	Sex/gender overall^3^	☐ basic^4^/ ☐ detailed^5^/ ☐ no information^6^ is provided regarding sex/gender aspects. ☐ Not applicable				

^1^ e. g., part-time/full-time working hours, female or male-dominated occupations/economic sectors, leadership, socioeconomic status, family work with unpaid household and carework.

^2^ or: Outcome data are analysed stratified by sex: results are checked for statistical significance.

^3^ Notes: were sex/gender aspects operationalised beyond the classification male/female?

^4^ Notes: sex and/or gender was assessed/ incl. explicitly (e. g., genes, hormones and/or gender-related life situations, behaviour)?

^5^ Notes: interactions between sex/gender are considered (e. g., interaction of coffee or alcohol consumption patterns and biological aspects).

^6^ Notes: no reflection is provided about the operationalisation of sex/gender.

In consensus discussions, we (MP, EB, KR, UL) adapted the checklist to observational study designs, such as cohort and case-control studies. For observational studies, the assessment of occupational exposure was added to the category “Study procedures of investigation or intervention and statistical analysis”. Although no cross-sectional studies met the inclusion criteria in the systematic review by Reichel et al. [23], it should also be possible to use the sex/gender checklist for cross-sectional studies [23,24].

Minimal modifications (Table 1: specifications were in bold) were made to the original checklist by Demetriou et al. (2019), regarding sex/gender-specific exposure or exposure assessment (e. g., Item 6b: study procedures **(assessment of occupational exposure** and/or intervention content and delivery, if appl.), to permit the assessment of sex/gender bias among observational studies of sedentary work. For example, the sex/gender checklist was expanded to encompass sex/gender in the description of physical activity/sedentary behaviour exposure in the study background to meet our basic requirements. For instance, van der Ploeg et al. [25] described in their introduction that men and women differ in their sedentary behaviour, referring to Australian National Health Survey data [25]. Furthermore, in the “Study procedures” category, we added the “assessment of occupational exposure” to the study procedure item and specified that sex/gender-related confounders for occupational health (e. g., part-time/full-time working hours, family work with unpaid household and carework) should be considered in the analysis (Table 1, specifications are in bold).

The reviewers independently rated each article using the sex/gender checklist by first assessing the level of reporting for all items using the following ratings: “detailed”, “basic”, “no information provided”, or “not applicable”. The reviewers then summarised the items for each category and assigned the highest score, which was achieved in one of two or two of three items, respectively, to a category. For example, if the first item in the background category was marked “no information” and the other two items were marked “basic”, then “basic” was selected for this category. To simplify the evaluation process, reviewers discussed the evaluation results together at the category level and followed the same procedure for determining the overall quality with which sex/gender was reported in each publication.

The overall score of a study corresponded to the highest score achieved in at least two of the four categories, with each of the four categories considered equally. For example, if “basic” was awarded in the categories “Background” and “Study design”, “no information” in the category “Study procedures” and “detailed” in the category “Presentation and interpretation of finding”, the primary study received an overall score of “basic”. A scoring of “detailed” in one category was insufficient to receive an overall score of “detailed”. In another example using the study by Møller et al. [26], the categories “Background and conceptual considerations” and “Study design” were marked “no information”, and the categories “Study procedures of investigation or intervention and statistical analysis” and “Presentation and interpretation of findings” were ranked “basic”. Thus, in the overall assessment, this study received the rating “basic” (S1 Table). If the overall assessment resulted in “no information”, there was a low consideration of sex/gender. The levels “basic” and “detailed” correspond to at least a fundamental consideration of sex/gender.

### Application of the sex/gender checklist and analyses

The adapted checklist was applied to the systematic review with a specific assessment of sex/gender bias. This was described in a PROSPERO protocol [24]. Among other things, this review aimed to summarise the research examining the association between occupational sitting and cardiometabolic risk and to determine whether the associations differ between men and women. The risk of bias was assessed using the Scottish Intercollegiate Guidelines Network (SIGN) checklist for cohort, case-control studies and controlled trials [17]. For the assessment of sex/gender bias, each publication was considered individually regardless of study affiliation (e. g., there were two publications from the Whitehall study), as the assessment of sex/gender bias may differ between publications even though the same population was considered.

First, two reviewers (KR, MP) piloted the sex/gender checklist on two cohort studies, two intervention studies, and one case-control study. Next, the two reviewers (KR, MP) independently rated each article using the sex/gender checklist. Finally, a third reviewer (EB) decided on all category-level and overall assessment disagreements. To examine the usability of the checklist, the reviewers also documented the average processing time of the checklist for each publication.

### Statistical analyses

Descriptive statistics were calculated for year of publication, risk of bias based on the SIGN, and study design stratified by overall sex/gender bias of the primary studies. We used bivariate and multivariable logistic regression models to examine the associations between consideration of sex/gender bias (*y*, dependent variable) and the publication characteristics (*x*_*i*_, independent variables), publication year, study design, and risk of bias based on the SIGN. The dependent variable is assigned the value 1 for at least a basic consideration of sex/gender bias (“detailed”, “basic”) and the value 0 for low or no consideration of sex/gender bias (“no information provided”). No study was rated as “not applicable” in the overall assessment (and this scenario is very unlikely), so the “not applicable” option was not included in the calculation. The model results are reported as odds ratios (ORs) with 95% confidence intervals (CIs). The multivariable regression model included all the variables. The variable for study design was categorised into intervention studies and observational studies (case-control and cohort studies) due to the sparsity of case-control studies. In a sensitivity analysis, we also considered publication year as a continuous variable. The risk of bias assessed with the SIGN was dichotomised in low and high risk of bias (reference). Statistical analyses were performed using IBM SPSS Statistics for Windows, version 29.0 (Armonk, NY: IBM Corp).

## Results

The adapted sex/gender bias checklist was pilot tested using the systematic review by Reichel et al. [23], which included 19 intervention studies, 25 cohort studies, and 5 case-control studies (39 studies described in 49 publications) in the data synthesis (S1 Table). Each reviewer needed an average of 20–30 minutes per study (reading and assessing) to complete the sex/gender checklist.

### Sex/gender checklist

**Fig 1:**
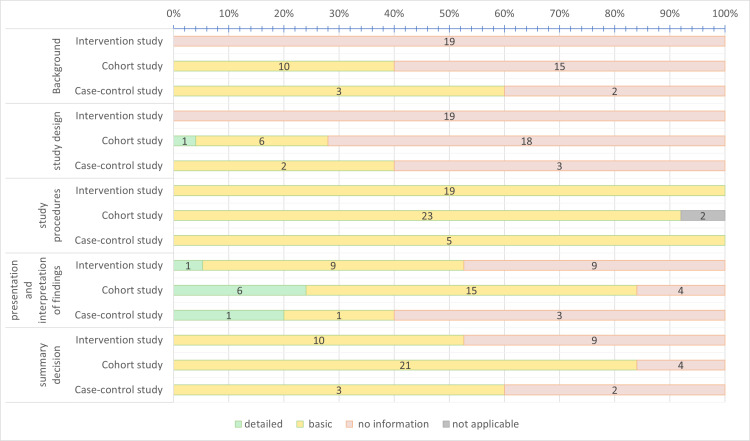
Distribution of primary studies (n = 49) in the respective categories stratified by study design.

In summary, 69% (n = 34) of the studies evaluated provided at least basic information and 31% (n = 15) provided no information regarding the consideration of sex/gender. Fig 1 shows the consideration of sex/gender stratified by study design in the four categories and the summary decisions.

#### Intervention studies.

None of the 19 intervention studies (Fig 1) described sex/gender aspects for the categories “Background and conceptual considerations” or “Study design”. All intervention studies described basic information for the category “Study procedures of investigation or intervention and statistical analysis”. However, this basic information comprised only a description of the number of men and women in the study population. Five intervention studies reported how sex/gender was considered in the statistical analyses [27–31]. In these statistical analyses, sex-adjusted results of four cluster randomised controlled trials [28–31] were presented and one study reported results stratified by sex [27]. This intervention study assessed sex/gender differences in the main outcome (interstitial glucose) [27].

#### Cohort and case-control studies.

Fig 1 shows that 60% (n = 15) of the cohort studies presented no information on sex/gender terminology, research questions or theoretical or conceptual linkages with sex/gender (“Background and conceptual considerations”) [26,32–45]. In 72% (n = 18) of the cohort studies, no information was given on measurement instruments or sample recruitment (“Study design”) [25,26,32,33,35,37–41,46–53]. Only one study provided detailed information in this category [54].

In 24% (n = 6) of the cohort studies, detailed sex/gender-related information was given for the category “Presentation and interpretation of findings” [33,36,42,43,48,49]. For example, Ferrario et al. [48] included only men, but discussed the results in the context of sex/gender. In ten (40%) cohort studies, a sex-stratified analysis was performed [33,35,36,42,43,45,49–51,55]. Sex-adjusted statistical association analyses were conducted in 13 (52%) cohort studies [26,32,34,37–39,42,44,45,49,51,52,56].

In three (60%) of the five case-control studies, no sex/gender information was provided in the “Study design” [57–59] or “Presentation and interpretation of findings” [57–59] categories (Fig 1). All case-control studies considered components of sex/gender analysis in the presenting of findings, for example, by reporting the number of men and women or by conducting different analyses for men and women. In two case-control studies, a sex-stratified analysis was performed [60,61]. Sex-adjusted association analyses were conducted in three case-control studies [58,60,61]. In the “Presentation and interpretation of findings” category, we found detailed information on differences between sex/gender in relation to the reported primary outcome (acute myocardial infarction) in one (20%) of the case-control studies [60].

### Associations between sex/gender bias and study characteristics

According to the bivariate and multivariable models, there was no statistically significant association between publication year as a linear variable and the consideration of sex/gender (bivariate model: OR per year = 0.89; 95% CI: 0.65, 1.23, multivariable model: OR per year = 0.98; 95% CI: 0.70, 1.38). The effect estimates for publication year categories from the bivariate and multivariable models were also not statistically significant (Table 2). The bivariate model showed that sex/gender tended to be considered less frequently in 2015–2016 (OR = 0.80; 95% CI: 0.17; 3.37) and 2017–2018 (OR = 0.56; 95% CI: 0.12; 2.54) than in the reference years (2012–2014). However, in the multivariable model, the odds that sex/gender was considered reversed for 2015–2016 compared to the reference years (2012–2014) (OR = 1.15; 95% CI: 0.19; 6.84).

**Table 2:** The effect of including potential confounding factors on the odds ratio (OR) for at least a basic/detailed consideration of sex/gender in articles (n = 49).

	No. of studies	Basic/Detailed	Basic model 1^a^		Adjusted model 2^b^	
	*n (%)*	*n (%)*	*OR*	*CI 95%*	*OR*	*CI 95%*
	49 (100)	*34* (100)				
*Year of Publication*						
2012–2014	16 (32.7)	12 (35.3)	1	–	1	–
2015–2016	17 (34.7)	12 (35.3)	0.80	0.17, 3.73	1.15	0.19, 6.84
2017–2018	16 (32.7)	10 (29.4)	0.56	0.12, 2.54	0.82	0.16, 4.13
*Study design*						
Intervention study	19 (38.8)	10 (29.4)	1	–	1	–
Observational study	30 (61.2)	24 (70.6)	3.60	1.01, 12.81	4.29	1.06, 17.38
*Risk of Bias* ^c^						
high RoB	39 (24.5)	28 (82.4)	1	–	1	–
low RoB	10 (20.4)	6 (17.6)	0.60	0.14, 2.50	0.34	0.06, 1.97

**OR,** odds ratio; **CI,** confidence interval; **n,** number of primary studies.

^a^Basic Model 1: bivariate (3 separate models) including publication year, study design, or risk of bias as the independent variable.

^b^Adjusted Model 2: multivariable model including publication year, study design, and risk of bias (RoB) as independent variables.

^c^assessed with the SIGN.

Compared to those in the intervention studies (reference), the odds of considering sex/gender increased by a factor of 3.60 (95% CI: 1.01, 12.81) in the observational studies. According to the multivariable model, the odds ratio of considering sex/gender increased by a factor of 4.52 (95% CI: 1.03, 19.91) in the observational studies. A low risk of bias (based on the SIGN) was not associated with at least a basic consideration of sex/gender in either the bivariate or the multivariable models (Table 2).

## Discussion

We demonstrated the application of a sex/gender checklist for the determination of consideration of sex/gender in aetiological studies in the field of occupational health within a systematic review.

We found that all studies (n = 49) examining occupational sedentary behaviour and cardiometabolic health outcomes included basic information regarding sex/gender in the category “Study procedures of investigation or intervention and statistical analysis”, and a few studies (n = 8) presented detailed information on sex/gender in the category “Presentation and interpretation of findings”. We found deficits among the intervention studies, as no intervention study (n = 19) presented information in the categories “Background and conceptual considerations” and “Study design”, and only 1 of the 19 (6%) intervention studies reported sex-stratified analyses. Cohort studies were more likely to provide detailed (6 of 23 cohort studies) or basic (15 of 23 cohort studies) sex/gender information for the “Presentation and interpretation of findings” than either case-control (1 detailed and 1 basic of 5 case-control studies) or intervention studies (1 detailed and 9 basic of 19 intervention studies). Observational studies were more likely to consider sex/gender than intervention studies. This might be because observational studies will almost always need to consider sex/gender as a confounding factor, whereas in RCTs it is expected that randomisation will remove most confounding. This may have led observational studies to consider and report sex/gender factors more thoroughly. Our finding that observational studies were more than three times more likely to consider sex/gender than intervention studies comprised a relevant difference that should be examined further in future studies. This suggests that sex/gender is currently not well addressed in occupational intervention studies of sedentary work.

Regarding our research questions, we found no temporal trend and no statistically significant association between risk of bias (based on the SIGN) and the consideration of sex/gender.

We expected an increase in consideration of sex/gender over time with growing awareness, however, this was not the case. Possibly, improvements in reporting began before our first included article was published in 2012. In 2000, Niedhammer et al. published a review of publications in occupational health epidemiology from six journals published in 1997 and reported that 11% of the 348 articles reviewed did not specify the sex of the study population, that women were still studied less often than men, and that sex was not investigated as a factor in many mixed-sex studies [62]. In 2022, Antequera et al. reported that overall sex/gender consideration in Cochrane reviews was inadequate [63]. Only 2.7% (14/516) of systematic reviews reported sex/gender in all of the analysed categories (abstract, methods, description of results, analytic results and discussion) [63]. All of the publications (n = 49) we assessed reported the sex of the study population, and we found that 40% of observational studies conducted and reported the results of sex-stratified analysis. Although it is not always possible to stratify by sex/gender when one group is insufficiently represented, epidemiologists should examine whether the dimensions of both sex and gender are adequately reflected in their research design, measures, and interpretation [64]. This includes accounting for dimensions of biological and/or social sex that are relevant to the research questions and identifying possible direct or mediated causal pathways for sex/gender-specific factors [64]. Current developments in research on biological sex and other gender perspectives (e. g., gender identity) must be taken into account [6,64–66]. Bauer recommends distinguishing between the effects of several sex/gender measures (e. g., sex at birth, gender identity) as well as sex-specific and sex-associated factors in the data analysis [64].

According to the recommendations of the SAGER reporting guidelines, the results should be reported stratified by sex and gender [9]. To date, there is no specific guidance from the Cochrane on how to assess primary studies in terms of sex/gender. In the “Handbook for Systematic Reviews of Intervention”, Cochrane recommends for systematic reviews that, for example, biological differences between women and men that may influence the response to an intervention should be made clear in the discussion of the results in the review. This includes a description of whether the included studies included all or only some of these groups and whether significant subgroup effects were found [67]. The unclear handling of sex/gender could be a possible explanation for why primary studies with a low risk of bias (based on the SIGN) did not consider sex/gender aspects.

Since interventions should ideally be tailored to both sexes [68] or better to all sexes, studies should also analyse results for men and women separately so that sex/gender-sensitive aspects can be evaluated. In this way, specific prevention strategies can be developed, when necessary [13]. However, conducting subgroup analyses in systematic reviews is difficult when too few primary studies report sex/gender-stratified results. In their systematic review of intervention studies of sedentary behaviour in boys and girls, Vondung et al. [15] excluded all studies (n = 72) that did not explain how they dealt with sex and gender in the analysis or did not report adjusted or sex-stratified results from their meta-analysis and assessed the included studies (n = 67) with the original sex/gender checklist [15].

### Strengths and limitations of the sex/gender checklist

This sex/gender checklist as a first step in the development of systematic reviews to explicitly consider sex/gender in primary studies in order to differentiate and compare sex/gender bias between different study designs and epidemiological questions. The reviewers (KR, MP, EB) found the checklist was useable and it allowed an overall assessment or an assessment at category level. The application of the checklist has shown in which areas future developments and research may be possible. When revising the sex/gender checklist, it is essential to consider current recommendations and developments in epidemiological research. The multidimensionality of sex/gender [64] should also remain an important component of the abridged sex/gender checklist. A further limitation is that only the final consensus results of the assessments were documented electronically, which did not allow calculation of kappa values for interrater reliability. This should be taken into account when evaluating future versions of the sex/gender checklist.

Although the reviewers found the evaluation time (20–30 minutes per study) acceptable, this time requirement might discourage researchers with limited resources to consider an assessment of sex/gender bias. A further limitation of the current checklist, was that the current checklist does not yet provide any guidance for assessing studies where only one sex/gender was included [46–48,53]. Guidance on how to rate such studies would be helpful, because occupational studies sometimes exclude the underrepresented sex/gender when researching occupations comprised predominantly of women or men (horizontal segregation) [69]. In addition, some studies may be designed to examine one sex or the other to fill the research gap created by previous sex/gender biased research. Andersson et al. [70], for example, conducted an intervention study of cardiovascular disease rehabilitation only among women to provide evidence lacking for women [70]. Dealing with this issue should be included in a revised sex/gender checklist. A conscious approach is essential to reflect the reasons for the inclusion or exclusion of gender-specific factors.

### Possible future developments/research

Horstmann et al. [6] included 77 different instruments in their scoping review and identified a temporal trend in the number of instruments between 2000 and 2020, and found four self-assessment instruments where participants classified themselves into one or multiple sex and/or gender categories. Even though the published survey instruments considering gender diversity increased from 2010 onwards [6], this was not reflected in the articles we assessed. Gender diversity and the handling is not yet sufficiently reflected in the sex/gender checklist. Future versions of the sex/gender bias checklist may need to be adapted to better assess gender diversity.

We see potential for optimising the sex/gender checklist better reflect how the consideration of sex/gender influenced the results of primary studies. This would require item-level analyses to determine which categories are most relevant or most strongly related with sex/gender-related results and conclusions. An item-level analysis may also show if it is possible to shorten the checklist without minimising its effectiveness.

The processing time of the current sex/gender bias checklist, while acceptable on its own, could be considered a cumbersome addition to the usual assessment of bias, especially when personnel resources and time are lacking or where many primary studies require assessment. A shortened version might be developed, for example one that could be integrated into other risk of bias tools for internal validity, such as those from Cochrane or the SIGN.

Further improvements could be made to how the items and categories are weighted in the summary rating. Currently, the item and categories are weighted equally. With the chosen liberal approach to study assessment studies that at least considered sex/gender basically were discriminated from those that did not. However, this blurred factor pertaining more to reporting quality with factors pertaining to methodological quality. For example, a study that does well with regard to “Background and conceptual considerations” may not have adequately accounted for sex/gender factors in the methods, which could have a greater influence on the results. For example, Jüni et al. [71] included 25 quality assessment scales for clinical trials in a meta-analysis and found that the use of summary scores made it difficult to identify high-quality clinical trials. They also showed that summary scores of quality assessment scale for clinical trials were not significantly associated with treatment effects [71]. To counteract these issues, the sex/gender checklist could be modified so that the items and categories are divided into major and minor domains that give more weight to factors more likely to impact the results. An examination of items or categories in major and minor domains and an improved algorithm for the overall assessment should be advantageous [72]. Also, further research with other examples from the field of occupational safety and health are needed.

## Conclusion

The sex/gender checklist for assessing the consideration of sex/gender in aetiological studies of occupational health showed that the sex/gender perspective is not sufficiently taken into account in primary studies of sedentary work behaviour and cardiometabolic health. Applying the checklist to an example systematic review demonstrated that the assessment is possible for different epidemiological study designs. Further improvements of the checklist, regarding its length and suitability for occupational research (e. g., how to assess studies excluding one sex/gender) are necessary. An optimisation of the algorithm for determining the overall sex/gender bias level is considered beneficial. Here, a clearer instrument without a total score and with a central focus on the sex/gender-specific evaluation would be required. Since the consideration of sex/gender has an impact on the interpretation of the aetiological results, as well as on the implementation of sex/gender sensitive prevention strategies, a suitable tool for assessing sex/gender bias is needed to encourage the deliberate consideration of sex/gender in research.

## Supporting information

S1 Table Quality of sex/gender bias in four categories and gender bias overall (stratified by study design). (DOCX)

## Abbreviations

SAGER: Sex and Gender Equity in Research
PROSPERO: International prospective register of systematic reviews
SIGN: Scottish Intercollegiate Guidelines Network
OR: Odds ratio
CI: Confidence interval
n: number of primary studies
n. a.: not applicable
RoB: Risk of bias
