# Supplementary material for: A tool for assessing sex/gender bias in epidemiological studies of occupational health: Pilot testing on studies of sedentary behaviour at the workplace and cardiometabolic health
Source: PLoS One. 2025 May 29;20(5):e0324391. doi: 10.1371/journal.pone.0324391 (PMC12121806; doi:10.1371/journal.pone.0324391)
Supplement: S1 Table — (DOCX) [file pone.0324391.s001.docx]

**S1 Table: Quality of sex/gender bias in four categories and gender bias overall (stratified by study design)**

| **Cohort studies (n = 25)** | | | | | | |
| --- | --- | --- | --- | --- | --- | --- |
| **No.** | **Study** | **Categories** | | | | **Coordinate decision** |
|  | **First author (year) references, type of population** | **Background** | **Study design** | **Study procedures**  **and**  **statistical analysis** | **Presentation and**  **interpretation of findings** | **Summary**  **Gender Bias** |
|  | Allesøe (2016) (46), Danish Nurse Cohort Study | + | 0 | n.a. | + | + |
|  | Allesøe (2015) (45), Danish Nurse Cohort Study | + | 0 | n.a. | + | + |
|  | Chau (2015) (31) , Trøndelag Health Study (HUNT3) | 0 | 0 | + | 0 | 0 |
|  | Eriksen (2015) (32), Danish Work Environment Cohort Study (DWECS) | 0 | 0 | + | ++ | + |
|  | Ferrario (2018) (47), Multinational Monitoring of Trends and Determinants in Cardiovascular Disease (MONICA), Pressioni Arteriose Monitorate E Loro Associazioni (PAMELA), and Surveillance of Employees of the Municipality of Milan (SEMM-Study) | + | 0 | + | ++ | + |
|  | Hall (2018) (52), Sister Study | + | 0 | + | + | + |
|  | Hayashi (2016) (48), Collaborative Cohort Study for Evaluation for Cancer Risk (JACC Study) | + | 0 | + | ++ | + |
|  | Johnsen (2016) (33), Swedish Work, Lipids and Fibrinogen (WOLF) study | 0 | + | + | + | + |
|  | Kikuchi (2015) (34) | 0 | 0 | + | + | + |
|  | Kim (2013) (35), Multiethnic Cohort Study (MEC) | 0 | + | + | ++ | + |
|  | Lin (2015) (49), National Longitudinal Survey of youth 1979 (NLSY79) | + | 0 | + | + | + |
|  | Martin (2013) (50), 1946 British Birth Cohort | + | 0 | + | + | + |
|  | Moe (2013) (36), Trøndelag Health Study (HUNT2) | 0 | 0 | + | + | + |
|  | Møller (2016) (24), Danish Work Environment Cohort Study (DWECS) | 0 | 0 | + | + | + |
|  | Picavet (2016) (37), Doetinchem Cohort Study | 0 | 0 | + | 0 | 0 |
|  | Pinto Pereira (2013) (51), 1958 British Birth Cohort | + | 0 | + | + | + |
|  | Pulsford (2013) (38), Whitehall II cohort study | 0 | 0 | + | + | + |
|  | Pulsford (2015) (39), Whitehall II cohort study | 0 | 0 | + | 0 | 0 |
|  | Saidj (2016) (40), Health 2006 cohort | 0 | 0 | + | 0 | 0 |
|  | Sakaue (2018) (44), Seven Countries Study | 0 | + | + | + | + |
|  | Smith (2017) (41), Canadian Community Health Survey (CCHS) | 0 | + | + | ++ | + |
| **No.** | **Study** | **Categories** | | | | **Coordinate decision** |
|  | **First author (year) references, type of population** | **Background** | **Study design** | **Study procedures**  **and**  **statistical analysis** | **Presentation and**  **interpretation of findings** | **Summary**  **Gender Bias** |
|  | Stamatakis (2017) (43), Whitehall II cohort study | 0 | + | + | + | + |
|  | Thompson (2017) (53) | + | ++ | + | + | + |
|  | van der Ploeg (2015) (23), Danish Work Environment Cohort Study (DWECS) | + | 0 | + | + | + |
| **Case control studies (n = 5)** | | | | | | |
| **No.** | **Study** | **Categories** | | | | **Coordinate decision** |
|  | **First author (year) references, type of population** | **Background** | **Study design** | **Study procedures**  **and**  **statistical analysis** | **Presentation and**  **interpretation of findings** | **Summary**  **Gender Bias** |
|  | Cheng (2014) (59), INTERHEART China study | + | + | + | ++ | + |
|  | Held (2012) (60), INTERHEART study 52 countries worldwide | + | + | + | + | + |
|  | Kumar (2013) (56) | + | 0 | + | 0 | + |
|  | Ma (2017) (57) | 0 | 0 | + | 0 | 0 |
|  | Selim (2013) (58) | 0 | 0 | + | 0 | 0 |
| **Intervention studies (n = 19)** | | | | | | |
| **No.** | **Study** | **Categories** | | | | **Coordinate decision** |
|  | **First author (year) references, type of population** | **Background** | **Study design** | **Study procedures**  **and**  **statistical analysis** | **Presentation and**  **interpretation of findings** | **Summary**  **Gender Bias** |
|  | Alkhajah (2012) (69) | 0 | 0 | + | 0 | 0 |
|  | Brocklebank (2017) (26) | 0 | 0 | + | ++ | + |
|  | Danquah (2017) (27), Take a stand | 0 | 0 | + | + | + |
|  | Dunning (2018) (70) | 0 | 0 | + | 0 | 0 |
|  | Graves (2015) (71) | 0 | 0 | + | + | + |
|  | Goreman (2013) (72) | 0 | 0 | + | 0 | 0 |
|  | Haslam (2019) (73), Walking work wonders | 0 | 0 | + | 0 | 0 |
|  | Healy (2013) (74), Stand-up Victoria | 0 | 0 | + | + | + |
|  | Healy (2016) (75), Stand-up Victoria | 0 | 0 | + | 0 | + |
|  | Healy (2017) (29), Stand-up Victoria | 0 | 0 | + | 0 | + |
| **No.** | **Study** | **Categories** | | | | **Coordinate decision** |
|  | **First author (year) references, type of population** | **Background** | **Study design** | **Study procedures**  **and**  **statistical analysis** | **Presentation and**  **interpretation of findings** | **Summary**  **Gender Bias** |
|  | Lin (2018) (76) | 0 | 0 | + | + | + |
|  | Mac Ewen (2017) (77) | 0 | 0 | + | 0 | 0 |
|  | Mailey (2016) (78), Beat the Seat | 0 | 0 | + | + | + |
|  | Mainsbridge (2014) (79) | 0 | 0 | + | + | + |
|  | Maylor (2018) (80) | 0 | 0 | + | 0 | 0 |
|  | Pesola (2017) (81) | 0 | 0 | + | + | + |
|  | Puig Ribera (2015) (82) | 0 | 0 | + | + | + |
|  | Verweij (2012) (28) | 0 | 0 | + | + | + |
|  | Zhu (2018) (83) | 0 | 0 | + | 0 | 0 |
| Legend: ++ “detailed information” of sex/gender; + “basic information” of sex/gender; 0 “no information of sex/gender provided”; n.a. = not applicable | | | | | | |
